# Microstructural damage of the cortico-striatal and thalamo-cortical fibers in Fabry disease: a diffusion MRI tractometry study

**DOI:** 10.1007/s00234-020-02497-7

**Published:** 2020-07-22

**Authors:** Sirio Cocozza, Simona Schiavi, Giuseppe Pontillo, Matteo Battocchio, Eleonora Riccio, Simona Caccavallo, Camilla Russo, Teodolinda Di Risi, Antonio Pisani, Alessandro Daducci, Arturo Brunetti

**Affiliations:** 1 Department of Advanced Biomedical Sciences, University “Federico II”, Naples, Italy; 2 Department of Computer Science, University of Verona, Verona, Italy; 3 National Research Council of Italy (IRIB CNR), Institute for Biomedical Research and Innovation, Palermo, Italy; 4 Department of Public Health, Nephrology Unit, University “Federico II”, Naples, Italy; 5 CEINGE - Advanced Biotechnologies, Naples, Italy

**Keywords:** Fabry disease, Magnetic resonance imaging, Brain, Tractometry

## Abstract

**Purpose:**

Recent evidences have suggested the possible presence of an involvement of the extrapyramidal system in Fabry disease (FD), a rare X-linked lysosomal storage disorder. We aimed to investigate the microstructural integrity of the main tracts of the cortico-striatal-thalamo-cortical loop in FD patients.

**Methods:**

Forty-seven FD patients (mean age = 42.3 ± 16.3 years, M/F = 28/21) and 49 healthy controls (mean age = 42.3 ± 13.1 years, M/F = 19/28) were enrolled in this study. Fractional anisotropy (FA), axial (AD), radial (RD), and mean diffusivity (MD) maps were computed for each subject, and connectomes were built using a standard atlas. Diffusion metrics and connectomes were then combined to carry on a diffusion MRI tractometry analysis. The main afferent and efferent pathways of the cortico-striatal-thalamo-cortical loop (namely, bundles connecting the precentral gyrus (PreCG) with the striatum and the thalamus) were evaluated.

**Results:**

We found the presence of a microstructural involvement of cortico-striatal-thalamo-cortical loop in FD patients, predominantly affecting the left side. In particular, we found significant lower mean FA values of the left cortico-striatal fibers (*p* = 0.001), coupled to higher MD (*p* = 0.001) and RD (*p* < 0.001) values, as well as higher MD (*p* = 0.01) and RD (*p* = 0.01) values at the level of the thalamo-cortical fibers.

**Conclusion:**

We confirmed the presence of an alteration of the extrapyramidal system in FD patients, in line with recent evidences suggesting the presence of brain changes as a possible reflection of the subtle motor symptoms present in this condition. Our results suggest that, along with functional changes, microstructural damage of this pathway is also present in FD patients.

## Introduction

Fabry disease (FD) is a rare X-linked lysosomal storage caused by an incomplete catabolization and subsequent intracellular accumulation of the glycosphingolipid globotriaosylceramide (Gb3), due to the defective activity of the α-galactosidase A (α-GalA) enzyme [1]. The unmetabolized glycosphingolipid therefore accumulates in different tissues, including the heart, kidney, and central nervous system (CNS), leading to the development of clinical symptoms [1]. With reference to CNS involvement, FD has been long considered to be a condition characterized only by major cerebrovascular events [2]. Nevertheless, a subclinical although significant impairment of motor functions, which occurs independently from cerebrovascular involvement, is present in FD patients, characterized by the presence of poorer fine manual dexterity, slower gait, and reduced hand speed [3].

In line with these clinical findings, recent advanced MRI studies have showed the presence of a deeper and complex brain involvement in FD patients, with particular reference to the motor system [4]. Indeed, an alteration of the corticostriatal pathway has been described in FD patients, and a reduced functional connectivity between the motor cortex and the striatum has been described in this condition [5]. An additional evidence of the involvement of the extrapyramidal pathway in FD has been demonstrated in a recent study showing the presence of susceptibility and volumetric alterations affecting two of the main relay stations of the extrapyramidal system, namely the striatum and the substantia nigra [6].

Although widespread microstructural alterations of the white matter (WM) are known to occur in FD patients, as demonstrated by different diffusion tensor imaging (DTI) studies [7–9], to date, no information about the integrity of the cortico-basal ganglia motor loop fibers is available. Given this background, the aim of this study was to evaluate the microstructural integrity of the main afferences and efferences of the motor cortices to the basal ganglia motor loop in FD patients, to investigate the possible presence of structural connectivity changes in these connections, and to expand the current knowledge about motor involvement in this condition.

## Material and methods

### Participants

In this retrospective cross-sectional study, part of a larger monocentric framework on the CNS involvement in FD, genetically proven patients were included along with age- and sex-comparable healthy controls (HC). For both groups, we evaluated male and female subjects without age limitations, with the following exclusion criteria: left handedness, co-existence of other systemic conditions or any addiction, history of stroke, head trauma, or any other clinical diagnosis of diseases affecting the CNS.

For all FD patients, clinical variables of systemic organ involvement were obtained from medical records and included the following: diabetes mellitus, hypertension, cardiac arrhythmia, left ventricular hypertrophy, renal failure (for estimated glomerular filtration rates < 90 mL/min), proteinuria (for scores > 150 mg/24 h), cephalalgia, and acroparesthesia.

The study was conducted in compliance with the ethical standard and approved by the “Carlo Romano” Ethics Committee for Biomedical Activities; written informed consent was obtained from all subjects according to the Declaration of Helsinki.

### MRI data acquisition

All subjects underwent an MRI scan on the same 3T scanner (Trio, Siemens Medical Systems, Erlangen, Germany), equipped with an 8-channel head coil. The MRI protocol included the following sequences: (a) 3D fluid attenuated inversion recovery (FLAIR): 160 slices; TR = 6000 ms, TE = 396 ms, TI = 2200 ms; voxel size = 1.0 × 1.0 × 1.0 mm^3^; (b) 3D T1-weighted: 160 slices; TR = 1900 ms, TE = 3.4 ms, TI = 900 ms, flip angle = 9°, voxel size = 1.0 × 1.0 × 1.0 mm^3^; (c) diffusion-weighted spin echo: TR = 7400 ms, TE = 88 ms, flip angle = 90°, voxel size = 2.2 × 2.2 × 2.2 mm^3^ with 64 directions at b = 1000 s/mm^2^ in addition to nine b = 0 s/mm^2^.

### MRI data analysis

For FD patients, T2-weighted hyperintense WM lesions were segmented (when present) by an observer with more than 8 years of expertise in neuroimaging data analysis, unaware of subject identity, employing a semi-automated technique (Jim 7; http://www.xinapse.com/home.php). From the segmentation procedure, lesion loads were obtained as an index of macroscopic WM damage. Furthermore, to correct for the potential impact of WM lesions in the subsequent analyses, the corresponding lesion masks were co-registered using an affine registration to the T1-weighted volumes for an in-painting procedure, as implemented in FSL, version 5.0.10 (FMRIB Software Library; http://www.fmrib.ox.ac.uk/fsl), by filling the mask with the mean signal intensity values of the surrounding normal-appearing WM.

For all subjects, the T1-weighted volumes were segmented using the standard FreeSurfer Desikan-Killiany atlas [10] which allowed to obtain a cortical parcellation of gray matter (GM) in 85 different regions of interest (ROIs). The intracranial volume (ICV) was also calculated as the sum of GM, WM, and cerebrospinal fluid volumes, in order to correct for differences in head sizes, which are known to occur in this condition [11].

DTI data were pre-processed to correct for motion and eddy currents [12]. Standard DTI metrics of fractional anisotropy (FA), axial diffusivity (AD), radial diffusivity (RD), and mean diffusivity (MD) were computed [13] using MRtrix (http://www.mrtrix.org). To perform global anatomically constrained tractography [14] (ACT), we first co-registered the T1 and DT images using FLIRT [15] (FSL, https://fsl.fmrib.ox.ac.uk) with boundary-based cost function [16]. Then, we computed the fiber orientation distribution functions [17, 18] and generated 1 million streamlines using the iFOD2 [19] tractography algorithm from which, for each subject, we built the corresponding connectome using the FreeSurfer parcellation in 85 ROIs. From the connectomes, we extracted the bundles connecting the precentral gyrus (PreCG) with the striatum (computed as caudate nucleus plus putamen) and with the thalamus, respectively, reflecting the main afferent and efferent pathways of the motor circuit within the cortico-striatal-thalamo-cortical loop [20, 21], as well as the corticospinal tract (CST) as a representation of the pyramidal system. Thus, we built a smaller connectome using only these as ROIs (three for the left and three for the right hemisphere).

Finally, DTI metrics and connectomes were combined to carry on diffusion MRI tractometry [22], which consists in assigning to each bundle a value that is obtained by taking the mean of the chosen metric along the streamlines composing the bundle.

**Fig. 1:**
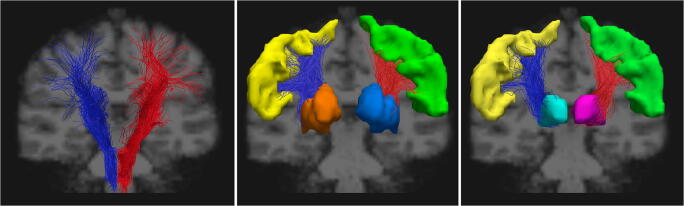
Image showing the reconstructed tracts in a 29-year-old female healthy control. From left to right, the cortico-spinal, the cortico-striatal and thalamo-cortical tracts (red indicates the left side, blue the right side), with the green and yellow areas indicating the left and right precentral gyri, respectively. Finally, the dark blue and purple regions of interests represent the left striatum (as the sum of caudate nucleus and putamen) and the thalamus, while in orange and light blue are displayed the contralateral regions

An example of the reconstructed tracts is available in Fig. 1.

### Statistical analysis

Statistical analysis was carried out using the Statistical Package for the Social Sciences package (SPSS, Version 23, IBM, Armonk, New York). Differences in terms of age and sex were tested using a two-sample *t* test and a chi-squared test, respectively. A general linear model (GLM) was employed to compare the two groups in terms of the diffusion MRI tractometry values derived from each DTI metric (FA, MD, AD, RD) on both hemispheres (left and right), including age, sex, and ICV as covariates, to remove the effects of potential confounding factors not related to microstructural damage. For each DTI metric, the corresponding mean value averaged over the entire WM volume was also included in the GLM as a nuisance variable, in order to correct for an index of global WM microstructural damage.

Results were considered significant for *p* < 0.05.

## Results

Forty-seven FD patients and forty-nine HC were included in this study, with the two groups being not different neither for age (*p* = 0.99) nor sex (*p* = 0.10). A complete list of the demographic and clinical information of the included population is available in Table 1.

**Table 1:** Complete list of the demographic and clinical information of the included population

	HC	FD
Age (mean ± SD)	42.3 ± 16.3	42.3 ± 13.1
Sex (M/F)	28/21	19/28
ERT	n.a.	35/47
ERT duration (mean ± SD)	n.a.	33.1 ± 30.6
Cephalalgia	n.a.	6/47
Acroparesthesia	n.a.	7/47
Hypertension	n.a.	12/47
Diabetes	n.a.	1/47
Arrhythmia	n.a.	3/47
Left ventricular hypertrophy	n.a.	23/47
Renal failure	n.a.	12/47
Proteinuria	n.a.	18/47

Subjects demographic and clinical variables of all subjects included in the study. Age is expressed in years, while ERT duration is expressed in months

*FD* Fabry disease, *SD* standard deviation, *ERT* enzyme replacement therapy, *n.a.* not applicable

**Fig. 2:**
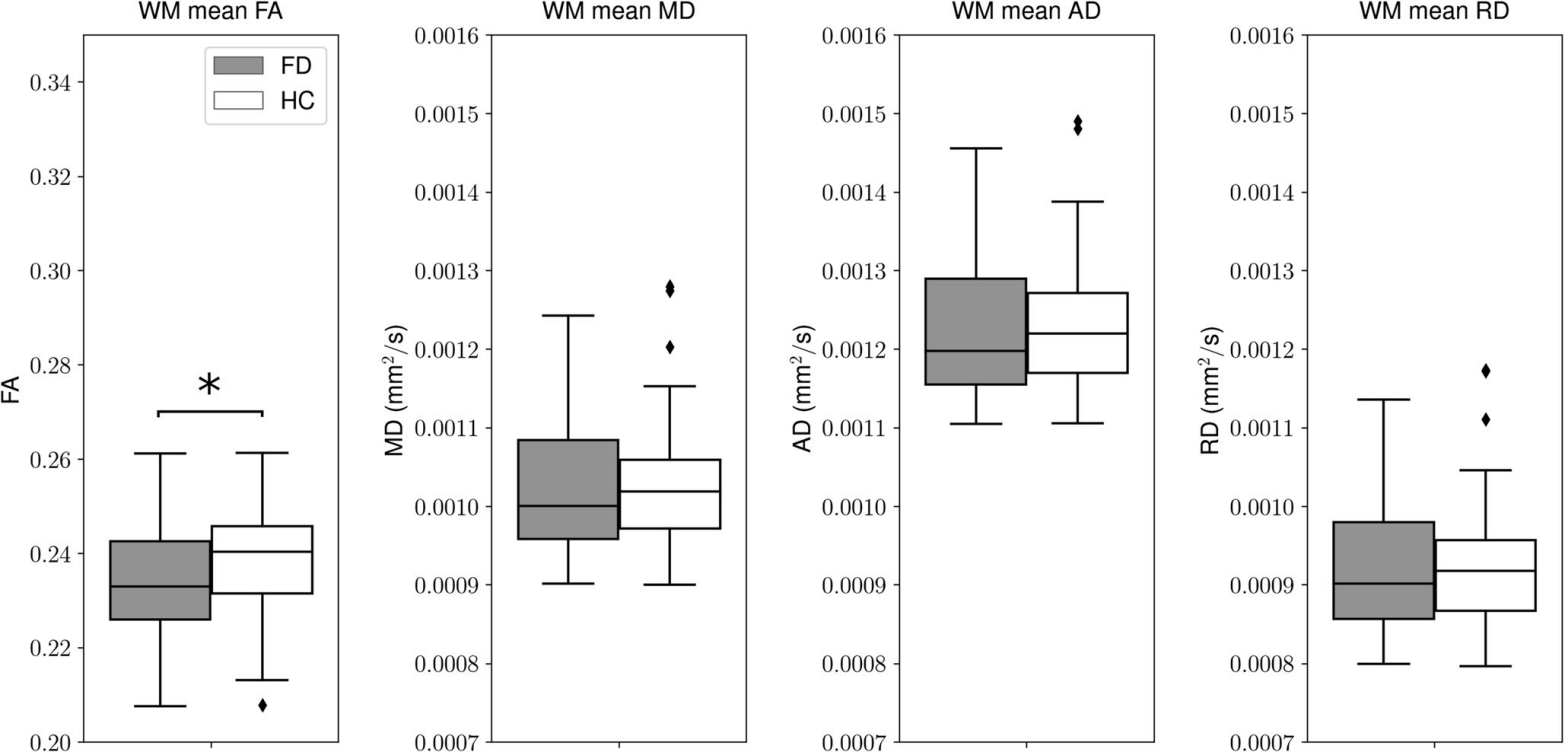
Box and whiskers plot showing the differences in terms of mean microstructural values along the entire white matter in Fabry patients compared with healthy controls. Asterisk indicates significant differences between the two groups

**Fig. 3:**
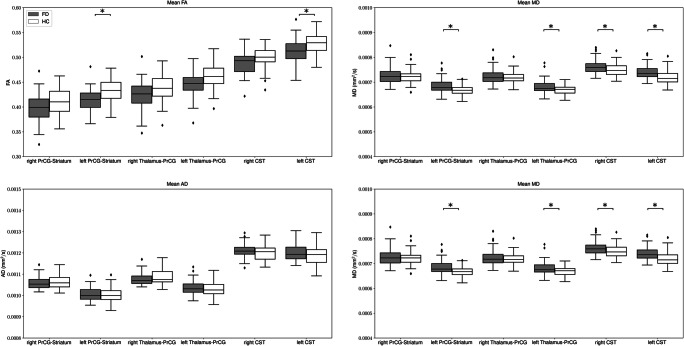
Box and whiskers plot showing the results of the tractometry analyses, with mean diffusion metrics along the evaluated tracts in Fabry patients compared with healthy controls. Asterisk indicates significant differences between the two groups

We found a diffuse microstructural damage of the entire WM highlighted by the significant difference in FA between HC and FD (0.238 ± 0.011 vs 0.233 ± 0.012, *p* = 0.02) (Table 2) (Fig. 2), along with the presence of a microstructural involvement of cortico-striatal tracts in FD patients, predominantly affecting the left side compared with the contralateral (Table 3; Fig. 3). In particular, we found a significant reduction of mean FA values of the left cortico-striatal fibers (0.43 ± 0.02 vs 0.41 ± 0.02 for HC and FD, respectively, *p* = 0.001), coupled to an increase in MD (0.67 × 10^−3^ ± 0.02 × 10^−3^ mm^2^/s vs 0.68 × 10^−3^ ± 0.03 × 10^−3^ mm^2^/s, *p* = 0.001) and RD (0.50 × 10^−3^ ± 0.02 × 10^−3^ mm^2^/s vs 0.52 × 10^−3^ ± 0.03 × 10^−3^ mm^2^/s, *p* < 0.001) values, while no differences emerged when AD maps were evaluated (1.00 × 10^−3^ ± 0.03 × 10^−3^ mm^2^/s vs 1.01 × 10^−3^ ± 0.03 × 10^−3^ mm^2^/s, *p* = 0.1109). When evaluating cortico-striatal connection on the right side, a trend of reduced mean RD was found in FD patients compared with HC, not reaching the statistical significance (0.55 ± 0.03 vs 0.56 ± 0.04, *p* = 0.09), while no differences emerged for the remaining variables (*p* = 0.34, *p* = 0.16 and *p* = 0.14 for FA, MD, and AD, respectively). Similarly, the thalamo-cortical tracts showed a predominant microstructural damage in FD patients for the left side compared with the contralateral. In particular, we found a significant increase in MD (0.67 × 10^−3^ ± 0.02 × 10^−3^ mm^2^/s vs 0.68 × 10^−3^ ± 0.02 × 10^−3^ mm^2^/s, *p* = 0.01) and RD (0.49 × 10^−3^ ± 0.03 × 10^−3^ mm^2^/s vs 0.51 × 10^−3^ ± 0.03 × 10^−3^ mm^2^/s, *p* < 0.001) values, while no differences emerged when FA and AD maps were evaluated. None of the metrics showed significant differences in the right hemisphere.

**Table 2:** Results of the between groups analyses investigating the global WM microstructure

	HC	FD	*p*
FA	0.238 ± 0.011	0.233 ± 0.012	*0.023*
MD (10^−3^ mm^2^/s)	1.032 ± 0.082	1.020 ± 0.086	0.565
AD (10^−3^ mm^2^/s)	1.237 ± 0.083	1.122 ± 0.089	0.331
RD (10^−3^ mm^2^/s)	0.929 ± 0.082	0.921 ± 0.086	0.711

Mean values and standard deviations of the diffusion metrics (FA, MD, AD and RD) of the entire WM for the two groups of subjects. In the last column, the *p* values obtained comparing HC and FD using a GLM with age, sex, and ICV are reported (the significant difference is in italics)

*FA* fractional anisotropy, *MD* mean diffusivity, *AD* axial diffusivity, *RD* radial diffusivity, *HC* healthy controls, *FD* Fabry disease, *GLM* general linear model, *ICV* intracranial volume

**Table 3:** Results of the between groups analyses investigating WM microstructure along the investigated tracts

Diffusion metric	Tract	Side	HC	FD	*p*
FA	PrCG-striatum	R	0.413 ± 0.025	0.399 ± 0.028	0.337
		L	0.434 ± 0.024	0.412 ± 0.023	*0.005*
	Thalamus-PrCG	R	0.438 ± 0.027	0.425 ± 0.028	0.482
		L	0.462 ± 0.025	0.445 ± 0.025	0.183
	CST	R	0.499 ± 0.021	0.488 ± 0.022	0.277
		L	0.529 ± 0.021	0.510 ± 0.025	*0.037*
MD (10^−3^ mm^2^/s)	PrCG-striatum	R	0.723 ± 0.027	0.728 ± 0.034	0.162
		L	0.668 ± 0.019	0.685 ± 0.029	*0.001*
	Thalamus-PrCG	R	0.720 ± 0.024	0.724 ± 0.032	0.222
		L	0.669 ± 0.018	0.683 ± 0.024	*0.010*
	CST	R	0.749 ± 0.025	0.764 ± 0.026	*0.031*
		L	0.718 ± 0.026	0.740 ± 0.026	*0.002*
AD (10^−3^ mm^2^/s)	PrCG-striatum	R	1.065 ± 0.034	1.059 ± 0.030	0.785
		L	1.001 ± 0.037	1.006 ± 0.035	0.092
	Thalamus-PrCG	R	1.087 ± 0.035	1.080 ± 0.029	0.813
		L	1.030 ± 0.035	1.036 ± 0.030	0.089
	CST	R	1.202 ± 0.037	1.209 ± 0.034	0.307
		L	1.190 ± 0.044	1.200 ± 0.037	0.198
RD (10^−3^ mm^2^/s)	PrCG-striatum	R	0.551 ± 0.030	0.563 ± 0.040	0.098
		L	0.501 ± 0.021	0.525 ± 0.038	*0.0002*
	Thalamus-PrCG	R	0.536 ± 0.029	0.547 ± 0.039	0.169
		L	0.489 ± 0.021	0.507 ± 0.031	*0.014*
	CST	R	0.523 ± 0.027	0.541 ± 0.033	*0.018*
		L	0.483 ± 0.026	0.510 ± 0.031	*0.0004*

Mean values and standard deviations of the diffusion metrics (FA, MD, AD and RD) of the three WM tracts (PrCG-striatum, thalamus-PrCG, and CST) for the two groups of subjects. The *p* values obtained comparing HC and FD using a GLM with age, sex, ICV, and mean values of the metric in the entire WM are reported in the last column (significant differences are in italics)

*FA* fractional anisotropy, *MD* mean diffusivity, *AD* axial diffusivity, *RD* radial diffusivity, *HC* healthy controls, *FD* Fabry disease, *PrCG* precentral gyrus, *CST* cortico-spinal tract, *WM* white matter, *GLM* generalized linear model, *ICV* intracranial volume

Finally, when evaluating microstructural changes affecting the CST, a less pronounced lateralization was found, with results showing a similar pattern of involvement, although mainly bilateral (Table 3; Fig. 3).

## Discussion

In FD patients, we found that prominent microstructural damage of the major WM tracts is implicated in both the extrapyramidal and pyramidal motor systems.

Poorer motor performance compared with age-matched HC has been described in FD patients, mainly involving functional domains (e.g., gait and hand speed) related to the extrapyramidal system [3]. Along with the evidence from ex vivo studies of pathologic Gb3 accumulation in different neuronal populations including the substantia nigra [23, 24], recent neuroimaging studies demonstrated the occurrence of neurodegenerative phenomena in two of the main hubs within the cortico-striatal-thalamo-cortical motor loop (i.e., striatum and substantia nigra), as well as functional disconnection between the motor cortex and the basal ganglia in this condition [5, 6]. In conjunction with these previous evidences, our results may support the hypothesis of a primary neurodegenerative damage of the extrapyramidal system, occurring at least in part independently from micro- and macro-vascular pathologies.

Indeed, microstructural damage of the cortico-striatal and thalamo-cortical projections may result from mechanisms of retrograde [25] and anterograde [26] transneuronal axonal degeneration, respectively, caused by primary neurodegeneration of intermediate relay stations—mainly the substantia nigra—within the cortico-striatal-thalamo-cortical motor loop. In accordance with this speculation, similar alterations of DTI metrics have been demonstrated in the frontal WM of Parkinson’s disease (PD) patients [27–29]. On the other hand, other tractography studies on PD patients reported opposed DTI alterations (i.e., increased FA and reduced MD) of the motor cortico-striatal and thalamo-cortical tracts [30], while concordant evidences exist on the CST showing an increase of FA (and a parallel reduction of MD) associated to PD, suggesting a reorganization of these fibers possibly reflecting a compensatory increase in axonal density due to axonal sprouting [27, 31].

Furthermore, the prominent alteration of WM RD over AD showed by FD patients in our sample appears to suggest myelin damage rather than axonal degeneration [32–34], so that the observed alterations may actually reflect subtle ischemic demyelination of the investigated tracts resulting from vascular pathology [35, 36]. Indeed, a similar pattern of DTI metrics alterations is known to characterize both WM hyperintensities (WMH) and normal appearing WM (NAWM) of patients with cerebral small vessel disease (SVD) [37, 38]. In particular, several voxel-based DTI studies demonstrated that the occurrence of vascular parkinsonism is associated with more prominent microstructural damage of the bifrontal WM, the corona radiata, and the anterior limb of internal capsule, which are the main tracts involved in movement control [39–42]. Indeed, it has been hypothesized that SVD disrupts the structural integrity of WM tracts, including the corticostriatal and thalamocortical fibers, thereby reducing the influence of the basal ganglia on motor, premotor, and supplementary motor cortices [41]. This disconnection of the basal ganglia-thalamo-cortical circuit could possibly lead to subcortical atrophy, ultimately resulting in parkinsonism. Furthermore, SVD could also lower the threshold for developing parkinsonism symptoms, modifying the threshold for Lewy body pathology to become symptomatic [41]. In this light, a similar mechanism could be theorized for FD, in which widespread WM microstructural damage has been demonstrated, not sparing the major frontal WM projection tracts [7, 8], whose prominent involvement could make FD patients more prone to the development of symptomatic or even subclinical impairment of motor functional domains, mainly related to the extrapyramidal system.

The prominence of WM microstructural damage on the left side observed in our sample of FD patients reasonably relies on the hand dominance of the studied subjects. Indeed, an asymmetry in DTI metrics of major motor WM tracts is known to exist in HC, with higher anisotropy values in the dominant hemisphere [43–46]. Therefore, in a condition in which a widespread microstructural damage occurs, differences of DTI metrics are more likely to emerge on the dominant side. Furthermore, due to the higher level of activation and energy demand, motor WM tracts of the dominant hemisphere are theoretically more disposed to ischemic injury and excitotoxic mechanisms [47]. Nevertheless, future studies comparing right- and left-handed FD subjects, although challenging to perform given the relatively rarity of the disease and the percentage of left-handedness in the population [48], are warranted.

Whatever its origin, disruption of the cortico-striatal projection fibers may underlie the reduction of functional connectivity between the motor cortex and the basal ganglia observed in this condition [5]. Nevertheless, it is known that the relationship between structural and functional connectivity may not be straightforward, so that future dynamic effective connectivity resting-state fMRI studies are warranted in order to unravel the causal connection between motor cortex and striatum functional activation [49].

Based on these observations, the question remains as to in which proportion primary neurodegenerative phenomena and cerebrovascular damage contribute to the motor functional impairment observed in FD. To disentangle this issue, further studies are needed, possibly oriented toward the direct investigation of microstructural damage at the level of substantia nigra and nigrostriatal connections, whose alterations bear the potential to represent more specific markers of primary neurodegenerative parkinsonism [50–54]. Furthermore, the lack of clinical data also needs to be acknowledged as a limitation of our study. However, even if theoretically the correlation with clinical measures of motor impairment could have helped to elucidate the functional meaning of the observed WM alterations, it is known that neurological alterations in FD patients are mild [3] and thus hardly relate to findings of advanced brain MRI techniques [5, 6].

Although characterized by these limitations, our results confirm the presence of an extrapyramidal involvement in FD patients, showing the presence of microstructural changes significantly affecting the cortico-striatal pathway in this condition, further confirming the presence of a deep and complex involvement of motor circuits in FD.
